# Symptomatic Leishmaniasis in an Italian Segugio Dog from Northeastern Romania: A Case Report

**Published:** 2018

**Authors:** Oana Irina TANASE, Constantin DARABAN, Elena VELESCU, Dragos BOGHEAN, Florentina BOCANETI-DARABAN

**Affiliations:** 1. Dept. of Public Health and Molecular Biology Laboratory, Faculty of Veterinary Medicine, University of Agricultural Sciences and Veterinary Medicine Ion Ionescu de la Brad, Iasi, Romania; 2. Univet Clinic, Iasi, Romania

**Keywords:** Leishmaniasis, Segugio, Serology

## Abstract

Leishmaniasis is a zoonotic parasitosis caused by a diphasic protozoan of the genus *Leishmania*. The dogs are considered the main domestic reservoir of *L. infantum* and its transmission occurs mainly through sand flies. We report the case of a 10 yr old Italian Segugio dog in Mar 2016 from Iasi County-Moldova Region, northeastern Romania, referred to a private clinic with progressive weight loss, dermal lesions over the muzzle, foot pads and over the right and left tarsal joints. The dog was born in Torino, Italy and transferred to Romania, with a history of regular travelling between these two countries. The physical examination revealed multiple cutaneous lesions with alopecia together with polyarthritis, lymphadenopathies, fatigue and weight loss. Neither fever or nor diarrhea were observed. The serological test (enzyme-linked immunosorbent assay) showed a positive result for Leishmaniasis. Light microscopy of the stained smears prepared from popliteal lymph node puncture failed to identify the amastigotes. The infection was treated using pentavalent antimonial therapy for eight weeks and Allopurinol for eight months. After nine months follow-up the dog presented with an improved body condition and no signs of recurrence.

## Introduction

Leishmaniasis is a zoonotic parasitosis caused by a diphasic protozoan of the genus *Leishmania*. Currently, both in the New and Old World, there are at least 12 species of *Leishmania* known to infect animals. The main species that cause leishmaniasis across the European continent is *Leishmania infantum* (1). The parasite is transmitted by sand flies, mainly by the genera *Phebotomus.* The dogs are considered the main domestic reservoir of *L. infantum* (2). In Europe, the disease is considered endemic in the Mediterranean region although, more and more cases are diagnosed in non-endemic countries such as Hungary, Croatia or Bulgaria (3). The prevalence of canine leishmaniasis (CaL) reported in the endemic regions varies from 10% to 70% (2).

Romania has been considered a country with sporadic cases of CaL and data regarding this infection in dogs are scarce. The first cases of clinical autochthonous CaL were reported in 1934 in southern Romania and since then only a few imported canine cases were published from southern and eastern Romania. Eighty years later, a new case of autochthonous infection was reported in southern Romania (4–6). Moldova region is located in northeastern Romania and no cases of *Leismania* infection were reported in imported or autochthonous dogs. Nevertheless, the disease was confirmed in one human patient without an external traveling history, but with a recent journey in southern Romania (7).

To complete its full development, the *Leishmania* parasites must undergo two stages in different hosts: a stage as promastigotes that requires the presence of an intervertebral host and a stage as amastigotes that requires the macrophage cells system of a mammalian host (8).

Leishmaniasis in dog shows different clinical evolution depending on the host immune response: from subclinical infection due to the host adequate immune response mediated by CD4^+^ T-cells and usually has a self-limiting character, to a “non-self-limiting” and severe clinical disease, with decreased amount of CD4^+^ and CD8^+^ T- cells (9,10). Commonly, the leishmaniasis manifests as a systemic disease. The clinical signs of disease vary according to the affected organs and may include several dermatological and ocular manifestations, lymphadenopathy, splenomegaly, renal disease, weight loss, and other nonspecific clinical signs, commonly accompanied by a specific cellular and a decreased humoral immunoreactivity. In addition, the clinical features in severe disease are accompanied by renal disorders, as consequence of the glomerular deposition of the specific immune complexes, of which the glomerulonephritis and tubulointerstitial nephritis are the most prevalent (9,10).

Most veterinarian practitioners from Moldova region classify CaL as an exotic disease. Therefore, there is a high risk of underdiagnosing or miss diagnosing CaL (6). Considering these facts, the early investigation and diagnosis of CaL are of importance both for the animal’s life and for the human leishmaniasis control (11). Usually, diagnosis of *Leishmania* infection (in both animals and humans) is stated after clinical, epidemiological and laboratory tests (12). The most commonly used laboratory tests for the diagnosis of leishmaniasis prove the existence of anti-*Leishmania* antibodies using indirect immunofluorescence or enzyme immunoassay (ELISA) (13).

In the following report, we present the first imported case of canine leishmaniasis in Moldova region, Romania.

## Case Report

**Fig. 1:**
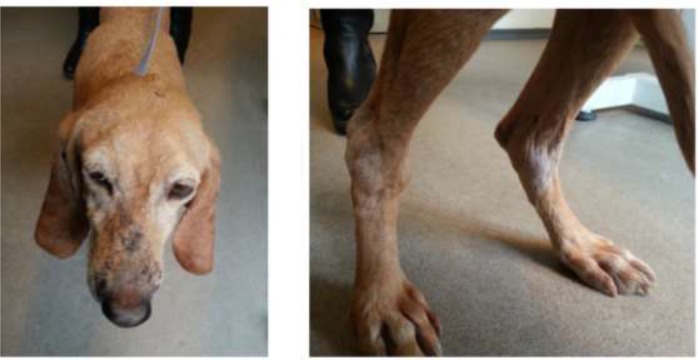
Clinical signs in a 10 yr old Italian Segugio dog with leishmaniasis: crusting dermatitis over the head (Left) and polyarthritis (Right)

In Mar 2016, a 10-yr-old Italian Segugio breed neutered female dog from Iasi County-Moldova Region, northeastern Romania, was presented at a local veterinary clinic. The dog was born in Torino, Italy and adopted from an animal shelter as a puppy by a Romanian owner and transferred to Romania. The female canine had a history of multiple backs and forward traveling from Romania to Italy. Symptoms at presentation were: progressive weight loss, skin wounds over the muzzle, foot pads and dermal lesions over the right and left tarsal joints. According to the owner, these lesions gradually appeared and progressed at least 30 d before the visit. On physical examination, multifocal alopecia and crusting dermatitis were seen (Fig. 1, Left) together with polyarthritis (Fig. 1, Right), lymphadenopathies, fatigue, and weight loss. No symptoms of fever or diarrhea were observed.

A direct radiography was performed. Furthermore, a blood sample was collected for serology and for complete blood count and serum biochemistry panel. The puncture of a popliteal lymph node was performed, for histological examination.

The enlargement of spleen and liver were seen, with no other changes. Considering the traveling history we followed the presumptive diagnosis of leishmaniasis. Serology for *Leishmania* spp. was submitted to the Synevovet Laboratory, Bucharest and performed by ELISA assay, providing a positive result, since the laboratory reference was considered negative. The blood count reported by the laboratory revealed mild leukopenia WBC-6.3×10^3^/mm^3^ (reference 6.9–12×10^3^), anemia – low hemoglobin level – 7.8 g/dl (reference15–29 g/dl) with low red blood cell count −3.9×10^6^ /mm^3^ (reference 5.50–8.50×10^6^ /mm^3^) and low hematocrit level −18.8% (reference 44%–57%), thrombocytopenia −117 ×10^3^/mm^3^ (reference 200–450 ×10^3^/mm^3^) and lymphopenia 0.5×10^3^/mm^3^ (reference 1–3.6 ×10^3^/mm^3^). Serum biochemistry showed hyperproteinemia 10.7 g/dL (reference 5.4–7.4 g/dL), low ala-nine transaminase level 6 U/L (8–57 U/L), low triglyceride levels 28 mg/dL (reference 37–39 mg/dL) and elevated creatine phosphokinase – 213 U/L (reference 14–120 U/L).

The puncture sample was submitted to the Department of Animal Pathology, Faculty of Veterinary Medicine of Iasi. Light microscopy of the stained smears prepared from popliteal lymph node puncture, failed to identify the amastigotes. However, a massive inflammatory reaction was seen, accompanied by lymphocytic and neutrophilic infiltration.

In this case, the diagnosis of CaL was based on the clinical symptoms, history of traveling to endemic area and on laboratory findings. The dog was treated using N-methylglucamine antimoniate (Glucantime) (50 mg/kg/BID subcutaneous) for eight weeks, and Allopurinol (10 mg/kg/BID per os) for eight months. After 9 months follow-up, the dog showed an improved general body condition, with no signs of recurrence. At this time, the blood count reported by the laboratory was in range.

The Ethics Committee of the university approved the study.

## Discussion

Canine leishmaniasis is a systemic zoonotic disease caused by the protozoan *Leishmania infantum*. Infected dogs are considered the main reservoir of the parasite (14) and play an important role in the epidemiology of human visceral and cutaneous leishmaniasis. Besides *Leishmania infantum, Leishmania tropica* is known to have important implications for both human and dog’s health, since this species was either isolated from cutaneous lesions and viscera of symptomatic dogs with leishmaniasis (15) and moreover from HIV-positive patients (16).

CaL is known to be endemic in more than 70 countries worldwide (17), especially in the Mediterranean areas (Italy, Malta, France, Spain, Portugal, Cyprus, Greece, Albania) (18), the Middle East (Iran) and many tropical and subtropical areas of the world (19, 20). Recently, many cases have been reported in non-endemic areas like the United Kingdom, Netherlands, Germany, and Poland as well as Eastern Europe - Croatia, Bulgaria, and Hungary. In these areas, the disease is still considered exotic by many practitioners (21, 1, 22). This is probably due to a wider spread of the vector sand flies, climate changing and more probably to a larger number of dogs being imported from or traveling to endemic countries (22).

For several decades Romania was presumed to have a non-endemic epidemiological status for this zoonotic disease. Our country is located at the northern border of sand fly distribution in Europe (18) and only sporadic cases were reported. Consecutively, recent studies performed in southern Romania revealed a prevalence of anti-*Leishmania* canine antibodies of 2.9% and all the dogs were clinically asymptomatic (5). This prevalence is restricted to a particular region. Therefore the general prevalence of the infected dogs living in Romania is unknown. No specific surveillance tests are required when animals from endemic areas are imported into the country. In endemic countries where the prevalence of the infection ranges from 5% to 60%, about 10%–30% of dogs become symptomatic (23). Whenever a dog presents a medium-high level of antibodies together with clinical signs, there is a strong suggestion of CaL infection (21).

In our case, the serological examination (ELISA) was able to detect the specific anti-*Leishmania* spp. antibodies, confirming the diagnosis. The enzymatic immunoassay is recommended as a first choice diagnostic method. Many studies showed a sensitivity of 91.8% and specificity of 83.8% for ELISA in diagnosing CaL (24). Interestingly, seropositivity is found in 88%–100% of dogs with physical signs and/or clinic pathological abnormalities consistent with CaL and only in less than 30% of the clinically healthy but infected animals (17). In fact, several studies showed the existence of resistant animals suffering from leishmaniasis with low parasite burden and reduced inflammatory responses, making it difficult to find parasites in the mononuclear phagocytic cells of the reticuloendothelial system organs (20). This observation can also apply to our case where we failed to detect amastigotes in the lymph nodes. Instead, we found a massive inflammatory reaction accompanied by lymphocytic and neutrophilic infiltration.

The incubation period for leishmaniasis may be variable, from months to years and in most of the cases, it depends on the host immunological response (22). In our case, the dog was born in Italy and had a history of multiple travels to this endemic area. Thus, the moment of infection was unknown. In this case, the dog was 10 yr old and given this history of traveling to endemic area, there was a great chance to become infected. Indeed, a higher seroprevalence is described in dogs greater than 8 yr old (25). Although *Leishmania* spp. infection is chronic disease specific for adult and old dogs, in some recent reports, the disease was diagnosed even in puppies (26).

This is first case of canine leishmaniasis reported in northeastern Romania, suggesting the possibility of a disease spread in Moldova region. We underline the necessity of a correct diagnosis and treatment of these cases in the non-endemic areas. Considering the absence of the vector, the parasite might be transmitted by blood transfusions, vertically from bitches to puppies or in a venereal manner.

We recommended a dual therapy association between N-methylglucamine antimoniate and allopurinol. These drugs are considered the standard therapy for CaL (27). The treatment proved to be efficient since at nine months follow-up, the dog showed an improved general body condition, with no signs of recurrence.

## Conclusion

Although the disease was reported for the first time in Moldova region, the veterinary practitioners should consider leishmaniasis in their differential diagnosis. Since dogs act as primary reservoir for human leishmaniasis, particular attention should be drawn to suspected cases that are returning from southern Europe with compatible clinical signs. Our report underlines the need for reliable diagnostic tests and specific treatment guidelines for leishmaniasis thus must be available in Moldova region.
